# Participatory Approach to Program Sustainment: Example From a Multisite National Geriatrics Telemedicine Program

**DOI:** 10.2196/82409

**Published:** 2026-03-27

**Authors:** Jessica T Riley, Eileen M Dryden, Camilla B Pimentel, Lauren R Moo, Meaghan A Kennedy, Steven R Barczi, Michelle I Rossi, Lynette R Kelley, William W Hung

**Affiliations:** 1 VA Bedford Healthcare System Center for Health Optimization and Implementation Research Bedford, MA United States; 2 Department of Population and Quantitative Health Sciences UMass TH Chan School of Medicine Worcester, MA United States; 3 New England Geriatric Research, Education, and Clinical Center (GRECC) VA Bedford Healthcare System Bedford, MA United States; 4 Department of Public Health University of Massachusetts Lowell Lowell, MA United States; 5 Department of Neurology Harvard Medical School Boston, MA United States; 6 Department of Family Medicine Boston University Chobanian & Avedisian School of Medicine Boston, MA United States; 7 GRECC William S Middleton Memorial VA Hospital Madison, WI United States; 8 University of Wisconsin School of Medicine and Public Health Madison, WI United States; 9 GRECC VA Pittsburgh Healthcare System Pittsburgh, PA United States; 10 Division of Geriatrics University of Pittsburgh Medical Center Pittsburgh, PA United States; 11 VA Eastern Colorado Health Care System Aurora, CO United States; 12 GRECC James J. Peters VA Medical Center Bronx, NY United States; 13 Icahn School of Medicine at Mount Sinai New York, NY United States

**Keywords:** telehealth, adaptation, maintenance, sustainment planning, aging, implementation science

## Abstract

**Background:**

Sustainment of evidence-based programs within dynamic health care environments requires ongoing adaptation to internal and external changes. Yet, strategies to support the sustainment of large-scale programs in heterogeneous settings are understudied. We developed and implemented a 3-phase participatory approach to support the sustainment of GRECC Connect, a 19-site Veterans Health Administration program that uses a hub-and-spoke model to expand rural access to geriatric specialty care.

**Objective:**

Our goal is to describe a novel participatory approach for identifying sustainment strategies for large-scale health care programs in complex environments, using our experience with GRECC Connect as an example to illustrate the application of this approach.

**Methods:**

We implemented the following 3-phase participatory approach with GRECC Connect team members from 19 hub sites. Phase 1: hub site clinicians and staff completed the Program Sustainment Assessment Tool, a publicly available online self-assessment of sustainability capacity. Phase 2: all sites then participated in a virtual retreat to exchange information, knowledge, and experiences related to sustainment strategies. Phase 3: each site submitted a locally-developed sustainment plan created with input from hub site team members. The sustainment plan worksheet included 3 questions asking respondents to reflect on the value of the participatory approach to sustainment. The process and experience of implementing this approach were also documented in structured meeting notes. Responses to Likert scale questions were analyzed with descriptive statistics, and qualitative data were analyzed using conventional content analysis.

**Results:**

Overall, there was a high level of participation across all 19 hub sites. In phase 1, a total of 25 individuals from 14 sites responded to the Program Sustainment Assessment Tool survey; in phase 2, a total of 58 individuals from 19 sites attended the retreat; and in phase 3, a total of 17 site sustainment plans were completed. Three primary sustainment paths were proposed and discussed during the retreat. Sites varied in their confidence to sustain program activities, but were able to articulate several barriers and facilitators specific to their site. The level of specificity in the sustainment plans varied considerably across sites. Most sites reported that this participatory approach was “very useful” (ie, ≥7 on a 10-point Likert scale) for planning their program sustainment.

**Conclusions:**

This approach offered a framework for sites to learn from one another, anticipate local barriers and facilitators, and move from reflection to identifying next steps for maintaining core program activities. Here, we describe the process used to guide 19 site teams through sustainment activities. We found the process is well-received, with sites reporting that their participation was useful for planning their sustainment journey. In elucidating our process, we provide a blueprint for other programs seeking to support sustainment across heterogeneous health care networks.

## Introduction

Sustainability has been defined as “the continued use of program components at sufficient intensity for the sustained achievement of desirable program goals and population outcomes” [1]. Many health care programs and services, even after being shown to be successful, are not sustained after their initial implementation period [2,3]. A wide array of program characteristics and organizational factors influence health care programs’ sustainability, including leadership support, consistent funding, adequate staffing, accountability, and role clarity [3,4]. Adding to the challenge, many have noted the need for programs to continually adapt to enhance their long-term sustainability amid ongoing internal and external contextual changes [5,6]. However, adaptation is complicated for large-scale, national health care programs that exist in heterogeneous settings. For programs operating across a system that includes varied settings, distinct contexts shape the program’s processes and details of service provision in unique ways that likely preclude the use of a singular sustainment strategy across sites.

Considering the myriad challenges facing health care (eg, COVID-19 pandemic, staff turnover, and decrease in budgets) in the United States [7], it is important to support the sustainment of evidence-based programs (EBPs) where appropriate to avoid the costs and burdens of implementing new, similar, or ineffective programs. Despite being recognized as a gap in the literature [8], strategies to support effective program sustainment remain understudied [1,3]. Work is needed to identify approaches that support the development of strategies to sustain effective health care services across networks [3,9]. Specifically, implementation science literature has recently called for research looking at EBPs in liminal periods of sustainment, meaning the time when a program has not yet been sustained or discontinued [9].

The Veterans Health Administration’s national GRECC Connect program is an example of an EBP that is in a liminal stage of sustainment. The GRECC Connect program, a multisite rural geriatric consultative network with nationwide reach, was developed as an innovation project to enhance access to geriatric care for rural veterans. As part of the planning for sustained program impact beyond the innovation phase, the national GRECC Connect leadership team initiated activities across multiple sites to identify potential sustainment paths. Prior descriptions of the GRECC Connect program reported differences across sites in their clinical setup, mainly reflective of the different context and needs of each site [10]. Given these differences, it seemed unlikely that a uniform, top-down prescriptive approach to sustaining GRECC Connect services would be successful [11]. To address this, we designed a participatory approach to support sustainment so each hub site could plan the most feasible and successful sustainment strategy for its own site.

We describe a 3-phase participatory approach used to support the sustainment of GRECC Connect that can serve as a template for planning the sustainment of other large-scale clinical innovations. Guided by the typology of sustainment approaches by Wolfenden et al [12], we label our approach “dynamic sustainment support,” which is defined as “a deliberate approach whereby external agencies provide resources or strategies to support [EBP] sustainment that continue to change over time….” due to program complexity or evolution. This contrasts with “self-sustainment” and “static sustainment” approaches included in typologies by Wolfenden et al [12]. As Wolfenden et al [12] noted, there is a need to characterize these approaches “to support sustainment and reflect on circumstances where they are most appropriate” to begin to address this gap of understanding in the literature [12]. To add to the limited literature on approaches to sustaining large-scale programs, we use our experience with the GRECC Connect program as an example to provide a robust description of a participatory approach to support program sustainment.

## Methods

### Case Example: Description of GRECC Connect

The GRECC Connect program, started in 2014 and funded by the Department of Veterans Affairs (VA) Office of Rural Health (ORH), connects geriatric interdisciplinary teams from 19 urban hub sites with older veteran patients in rural areas through secure VA-approved videoconferencing platforms [10]. Geriatric specialty clinicians at the hubs connect to patients who are either located in a rural community-based outpatient clinic (clinical video telehealth) or are in their home (VA Video Connect). This hub-and-spoke model delivers geriatric care based on the needs of primary care teams in rural areas and on the expertise available at the hub sites. The types of geriatric consultation services delivered by hub sites include the assessment or management of cognitive impairment, bone health and fall prevention, comprehensive geriatric assessments, and management of complex comorbidities (see Pimentel et al [10] for a full description of the GRECC Connect program).

GRECC Connect has demonstrated success at identifying unmet care needs, avoiding potentially inappropriate medications, and linking patients with needed care [10,13]. Patients and caregivers who have experienced GRECC Connect clinical encounters report numerous benefits (eg, high satisfaction and reduced caregiver burden) [14] aligned with standards for comprehensive geriatrics care [15]. While GRECC Connect providers were pioneers in providing telemedicine to older adults, since the COVID-19 pandemic, this practice has become more widespread [16,17]. However, the interdisciplinary nature of the service, focus on rural older adults, and the expertise built over almost a decade providing high-quality geriatric care online, make the GRECC Connect program unique.

GRECC Connect has been designated as a “promising practice” by ORH [18] and received “innovation” funding support, that is, special funding for the initial project development and the subsequent spread of the practice to multiple hospitals. Initially, GRECC Connect was developed and implemented by clinicians affiliated with several Geriatric Research, Education, and Clinical Centers (GRECCs) [19]. GRECCs are VA geriatric centers of excellence focused on aging that are located at medical centers across the country. They often act as incubators for new models of care. To be sustained long-term beyond the initial “innovation phase,” however, these GRECC Connect activities need to be integrated into established VA health care system services as part of routine care for veterans. This means the innovative processes that were developed and implemented specifically to increase access to geriatric specialty care telemedicine visits for rural patients would need to be intentionally incorporated into and supported by the existing system. Given the size and complexity of the US Veterans Health Administration network, there may not be a one-size-fits-all integration solution across all health care facilities. Integrating into the existing system could involve shifting how resources are allocated, hiring new staff, or requiring existing staff to expand their scopes of practice. In the absence of this effort, any gains achieved by this innovative program could be lost. For example, while telemedicine in general is likely to continue, the capacity to engage in geriatric telemedicine may decline, or the target audience (ie, rural veterans) may not be reached. In fall 2022, in anticipation of the innovation funding shifting, national GRECC Connect leadership initiated a process to identify strategies for sustaining GRECC Connect clinical services (ie, specialty geriatric telemedicine care) at the 19 hub sites.

**Figure 1:**
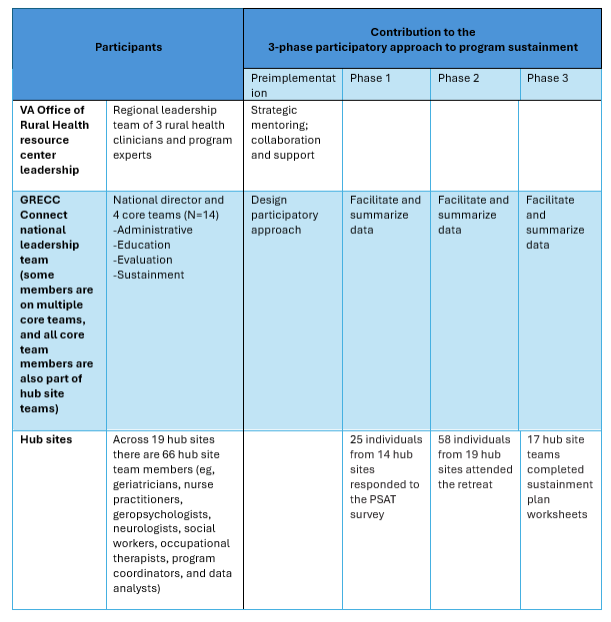
GRECC Connect example: participation in a 3-phase participatory approach to program sustainment. PSAT: Program Sustainment Assessment Tool; VA: Department of Veterans Affairs.

We leveraged the program’s already developed infrastructure and expertise in designing and carrying out the participatory process planning. GRECC Connect’s robust administrative structure consists of a national director and 4 “core” groups (Figure 1). These core groups help coordinate the activities of the 19 GRECC Connect site directors and their associated team members. Core group team members are a subgroup of staff from the 19 hub sites, many having volunteered to participate in more than one core group. These groups include (1) an evaluation core that has qualitative and quantitative subgroups and is charged with evaluating the program’s process and outcomes, (2) an education and workforce development core that focuses on developing and implementing a case conference series on geriatric care for rural providers, (3) a sustainment core that works within and across sites to guide on implementing GRECC Connect best practices and overcoming barriers to implementation, and (4) an administrative core charged with guiding and developing budgets and reports. The administrative work of GRECC Connect is moved forward through a series of online meetings: each core group meets monthly or quarterly, the leads of the 4 core groups meet monthly with the national director, and members of all 19 hub site teams join a monthly national GRECC Connect “all site” meeting.

The GRECC Connect Qualitative Evaluation Core team (QualEC) evaluates the program via interviews, surveys, and other qualitative methods. Members include 6 VA researchers with expertise in program evaluation, qualitative methods, implementation science, geriatric medicine, and primary care. Using the GRECC Connect administrative infrastructure described above, this QualEC team supported the development of a 3-phase participatory approach in collaboration with sustainment core leadership. The process and experience of implementing this approach were documented in structured meeting notes.

### Implementation of 3-Phase Participatory Approach to Program Sustainment: GRECC Connect Example

**Figure 2:**
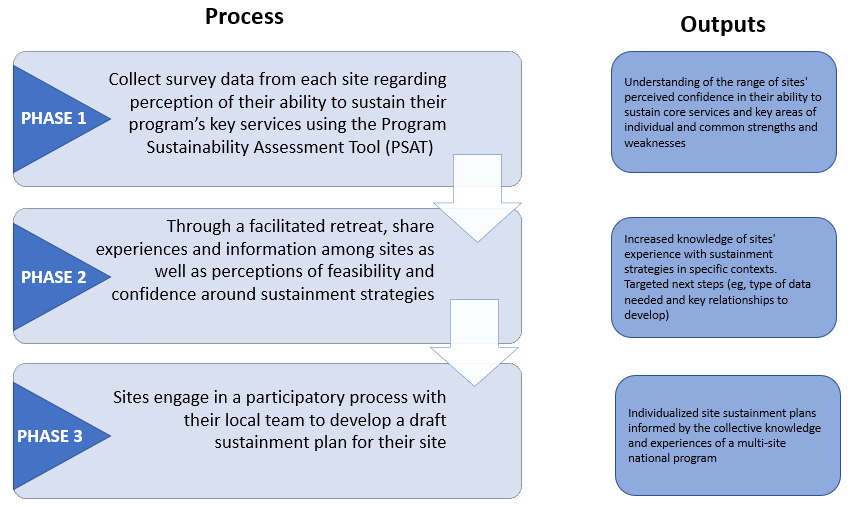
Process and outputs of the 3-phase participatory approach to program sustainment. PSAT: Program Sustainment Assessment Tool.

In phase 1, we established a baseline understanding of each hub site’s perception of the likelihood of sustainment of their GRECC Connect-related activities beyond the current period of funding support. In phase 2, we shared information about potential sustainment strategies and supported discussion among the sites to exchange experiences and knowledge and to help each site determine next steps in their planning process. Phase 3 involved encouraging site-level participatory processes by asking sites to complete a sustainment plan worksheet with input from all members of their local GRECC Connect team (Figure 2).

### Phase 1: Establishing a Baseline Understanding of Sites’ Perceptions of Sustainability

In spring 2023, we used the Program Sustainability Assessment Tool (PSAT [20]) to gauge sites’ perceived ability to sustain core GRECC Connect activities beyond the initial period of funding. The PSAT is a publicly available, online self-assessment tool that measures a program’s perception of its sustainability capacity. The 8 components measured in the PSAT include environmental support, funding stability, partnerships, organizational capacity, program evaluation, program adaptation, communications, and strategic planning. The scale for each domain ranges from 1 (no extent) to 7 (full extent). We emailed a link to the PSAT to team members at each of the 19 hub sites and requested that site clinicians and staff complete PSAT assessments within 1 week. Our goal was to begin a reflective and participatory process to help sites understand factors influencing future sustainability and to gauge areas of strength and weakness within their site. This also provided a baseline sustainability score for each site and insight into promising components to focus on to enhance their likelihood of sustainment, as well as what areas in which they may need support.

The PSAT data were exported into Excel (Microsoft Corp) for analysis. QualEC team members calculated the average and range for each of the 8 PSAT components and for the overall score across all sites. Where more than one PSAT was completed for a given hub site, we first averaged the responses within the site and then used the average in the cross-site analysis.

### Phase 2: Sharing Experiences and Information Among the National Group

#### Overview

We followed the PSAT administration with an opportunity for sites to share experiences and information about sustainment strategies. We believed a national discussion that included perspectives from multiple sites would be necessary to design sustainment strategies relevant to each site’s unique context. Some sites had more sustainment experience and expertise that would be valuable to share with the other sites. We extended a regularly scheduled monthly GRECC Connect all-site national meeting from 1 to 2 hours and used that time to host the online retreat using the Teams (Microsoft Corp) platform, organizing multiple breakout rooms to promote sharing by different roles and professions of staff participants.

#### Identification of Potential Sustainment Strategies

**Figure 3:**
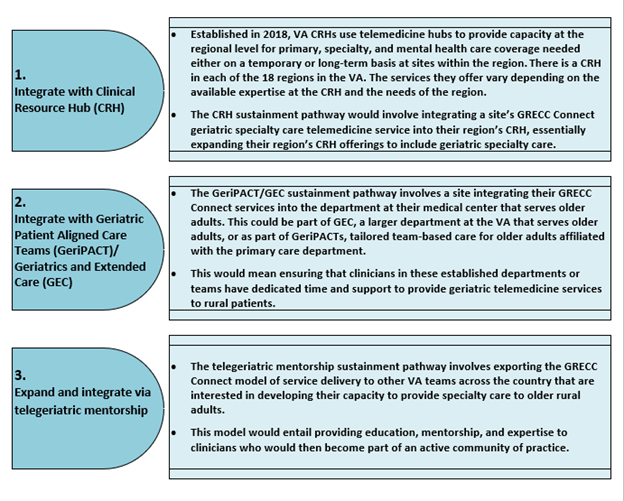
GRECC Connect example: sustainment strategy details. CRH: Clinical Resource Hub; GEC: Geriatrics and Extended Care; GeriPACT: Geriatric Patient Aligned Care Team; VA: Department of Veterans Affairs.

In collaboration with the GRECC Connect sustainment core, the QualEC developed the retreat agenda focusing on three potential sustainment strategies: (1) integrating services into a regional telemedicine hub that provides gap care coverage due to staffing shortages (the Clinical Resource Hub [CRH] [21] integration strategy); (2) integrating services into the teams or departments that currently serve older adults (the Geriatric Patient Aligned Care Team [GeriPACT] [22]/Geriatrics and Extended Care [GEC] integration strategy); and (3) exporting the model to other interested teams throughout the health care system through a mentoring hub (the telegeriatric mentorship strategy, see Figure 3 for details and further explanation). These strategies were previously identified and developed by the GRECC Connect core team leadership in consultation with our funders at ORH and with input from the field (ie, GRECC Connect team members from hub sites across the country) during one of the regularly scheduled national all-site meetings. Each potential model had previously been established within individual VA facilities or as a mechanism for supporting a national VA initiative. These proposed sustainment strategies were frameworks for sites to use to begin their sustainment process planning and helped identify ways in which sites may need to be supported.

#### Retreat Agenda

All GRECC Connect sites participated in the online retreat held in March 2023. The GRECC Connect national director shared the purpose of the retreat—to discuss how to continue bringing geriatrics interdisciplinary team care to older, rural patients—and the history of GRECC Connect and its status as an ORH Promising Practice. We shared sites’ anonymized PSAT scores to promote a common understanding of the range of perceptions of sustainability capacity. We then launched an electronic poll to assess each participant’s confidence that their site’s service to older, rural patients would be sustained beyond current ORH funding (on a scale of 0-10, 0=not at all). Before separating into predetermined “breakout” rooms, GRECC Connect site leads with knowledge on the 3 potential sustainment strategies gave a brief overview of each. After each overview, we launched 2 polls to assess, on a scale of 1-7 (1=not at all), participants’ perspectives on the feasibility and importance of the potential sustainment pathways for their site.

#### Breakout Rooms

We assigned the GRECC Connect hub site staff to 1 of 3 discussion groups focused on each strategy. Preassigning groups for each sustainment strategy allowed us to control group composition to encourage staff of varying backgrounds and site locations to join the discussion. For example, we purposefully distributed higher-level leadership and participants from sites with more expertise and experience among the breakout rooms. Preassigned groups also meant that GRECC Connect staff from various geographic sites, which have different operating contexts, would be able to share their knowledge and insights. The goal was to provide an environment that would enable sites to learn from one another.

Discussion within each of the 3 breakout rooms was guided by and adapted from the suggested exploration of the maintenance dimension of the RE-AIM (Reach, Effectiveness, Adoption, Implementation, and Maintenance) framework [23] by Shelton et al [6]: (1) How important is this strategy? Why or why not? (2) What could dissemination to the field look like? (list scenarios) and (3) What are barriers? What are the factors that may facilitate this?

Participants were given the same template to use in each breakout room, regardless of the strategy they were discussing. The template also guided how to facilitate the discussion and included space for notes. Participants discussed the strategy for 20 minutes. After the allotted time, attendees returned to the larger online platform, where each group’s assigned lead reported their small group discussion to the larger group.

The final component of the retreat was a second round of 20-minute breakout rooms. Participants selected their desired breakout room based on the preceding large group discussion and perceived usefulness for their own site. Participants used a template to guide discussion and identified interested parties relevant to their sustainment strategy, data that would be needed to support “buy-in” to the sustainment strategy, and 3-4 concrete next steps to move the strategy forward (see Multimedia Appendix 1 for a full list of questions used in the breakout rooms). As in the first breakout session, groups were assigned a lead who reported back to the larger group.

We collected the notes from each breakout group’s assigned lead and summarized them. We also exported and calculated descriptive statistics on the data from the 6 poll questions.

### Phase 3: Using National Collective Knowledge and Local Participation to Develop Tailored Site-Specific Sustainment Plans

#### Overview

The retreat output (ie, summarized qualitative discussion notes and quantitative poll results) guided our understanding of the type of support that was needed and the most useful next steps. After the retreat, the national program director met with each site to discuss ongoing administrative topics as well as their immediate postretreat thoughts on sustainment. He invited sites to join a weekly QualEC meeting to brainstorm ways of collecting data they needed to either inform the decision of what path to choose or to help them navigate their sustainment pathway of choice.

We created a “sustainment planning worksheet” for each site to complete and return within 2 months of receipt. Its purpose was to encourage sites to begin to think through their need for the GRECC Connect program and how they envisioned their individual program being sustained. The sustainment planning worksheet mimicked a brief grant proposal and included sections on background and rural significance, purpose and objectives, design and planned activities, key partners, measurement plan, products and deliverables, long-term planning, and potential barriers to their proposed sustainment plan (Multimedia Appendix 2). This process ensured that team discussions and thought exercises would occur with enough time to address any barriers that surfaced and to facilitate a planful transition. We emphasized the importance of including all GRECC Connect site team members when completing the worksheet. During this period, GRECC Connect continued to hold a regular monthly call, where leadership dedicated time on the agenda to answer questions staff had while completing the sustainment worksheet.

Soon after we distributed the sustainment planning worksheet, the team members at the GRECC Connect site that leads the QualEC group piloted the sustainment worksheet with all team members, documenting additional clarifying guidance that would be helpful to know as it was being completed. We shared the guidance with the national team and, once again, offered to meet individually with sites to help guide them as they worked through their site’s sustainment planning worksheet. We also distributed supportive documents to help sites pursue information about which sustainment pathways might best align with their sites’ needs and context, such as contact information for each region’s CRH director. Completed sustainment worksheets were reviewed in detail by members of the sustainment and evaluation core teams. Then, to facilitate comparison, responses to the worksheets were entered into an Excel template where each column was a question on the template and each row was a hub site. Qualitative data were analyzed using the conventional content analysis approach [24].

#### Evaluation of the 3-Phase Participatory Approach to Program Sustainment

The second part of the sustainment planning worksheet included a series of reflection questions about the participatory process itself. The reflection portion of the worksheet included 3 Likert scale questions followed by a space to add a comment. The reflection questions were as follows: (1) To what extent was your participation in the March 2023 Online GRECC Connect Retreat, as well as other site planning activities (discussions with other sites regarding sustainment strategies, self-assessment tools [PSAT]), useful for your sites’ planning? (Scale 0 to 10, 0=not useful at all); (2) To what extent was the process of completing this proposal useful for your sites’ planning? (Scale 0 to 10, 0=not useful at all); (3) How confident are you that your service to rural patients can be sustained using your proposed site sustainment strategy? (Scale 0 to 10, 0=not confident at all). An additional open-ended question asked, “Are there additional things that GRECC Connect leadership could do to support your sites’ sustainment planning?” Likert scale responses were aggregated into 3 categories (0-3=minimally useful, 4-6=useful, 7-10=very useful; 0-3=minimally confident, 4-6=confident, and 7-10=very confident, Multimedia Appendix 2). Responses to Likert scale questions were analyzed with descriptive statistics, and qualitative data were analyzed using conventional content analysis [24].

### Ethical Considerations

The VA Bedford Healthcare System Institutional Review Board determined this work was undertaken to inform VA operations as part of program evaluation and quality improvement activities and was not human participants research. The need for ethics approval was waived by the VA Bedford Healthcare System Institutional Review Board, which determined this program evaluation activity to be nonresearch. As a nonresearch activity, formal informed consent was not required; however, participants were informed about the project plans, including that participation was voluntary, they could opt out of being recorded, and all data collected would be deidentified for publication purposes. Under VA policy, no compensation is provided to VA staff for their participation in these activities.

## Results

### Implementation of Approach: GRECC Connect Example

#### Phase 1: Establishing a Baseline Understanding of Sites’ Perceptions of Sustainability

We received 25 individual PSAT responses across 14 (of 19) GRECC Connect sites; 6 sites had more than 1 respondent. Respondents included geriatricians, nurse practitioners, geropsychologists, neurologists, social workers, and occupational therapists (Figure 1).

Sites varied in their perceived confidence to sustain their GRECC Connect program. Overall sustainability scores across the 14 sites were wide ranging (mean 4.8, SD 0.9; range: 1-7). The “program adaptation” domain had the highest average rating across sites (mean 5.8, SD 0.7), while “partnerships” had the lowest average score (mean 3.4, SD 1.3). Respondent rated the other domains as follows: “environmental support” mean of 5.4 (SD 0.7), “funding stability” mean of 4.3 (SD 1.3), “organizational capacity” mean of 4.5 (SD 1.1), “program evaluation” mean of 5.4 (SD 0.9), “communications” mean of 4.5 (SD 1.4), and “strategic planning” mean of 4.6 (SD 1.2).

#### Phase 2: Sharing Experience and Information Among the National Group

A total of 58 (of 66) GRECC Connect hub site team members (eg, geriatricians, other clinicians, project coordinators, and administrative staff) from all 19 sites participated in the retreat (Figure 1).

When publicly polled, sites reported varying confidence in their ability to continue serving rural patients beyond current funding (mean of 5.12, SD 2.54; range of 0-10). Sites had mid-range perceptions of the importance and feasibility of the 3 sustainment strategies. Out of a maximum score of 7, the GeriPACT/GEC strategy was rated the most important (mean 4.9, SD 1.18) and most feasible strategy (mean 3.9, SD 1.43). The average importance of the CRH strategy was rated a 3.8 (SD 1.87) and the feasibility a 3.5 (SD 1.58) while the average importance and feasibility of the telegeriatric mentorship strategy was rated a 4.3 (SD 1.49) and 3.6 (SD 1.06), respectively.

Sites talked openly about anticipated barriers to sustainment and also potential facilitators. Guided by prompts, this led to strategy development that purposefully considered their local context, intentionally leveraging and addressing the facilitators and barriers. Some potential next steps identified by the GeriPACT/GEC small group included, for example, starting conversations between GeriPACT directors and local GRECC Connect directors about interest, feasibility, and logistics (eg, data collection or gap analysis that would start to support a business case for GeriPACT/GEC to support GRECC services).

#### Phase 3: Using National Collective Knowledge and Local Participation to Develop Tailored Site-Specific Sustainment Plans

After the retreat, 17 of the 19 sites completed and returned the sustainment proposal worksheet (Figure 1). Of those, 14 identified a primary sustainment path for their site; 11 chose at least 1 of the 3 paths presented, while 3 identified new, different sustainment paths for their site. One site indicated they required significant additional support to identify a sustainment strategy and plan for future implementation. The level of specificity included in the completed worksheets varied considerably. At one end of the spectrum, 7 sites submitted sustainment plans that were rigorous and thorough enough to guide their sustainment journey. On the other end of the spectrum, 4 sites submitted vague plans, lacking sufficient detail to act as a blueprint for implementation. The remaining plans demonstrated certain details, but with some steps needing further consideration.

### Evaluation of Approach

Sites reported that participation in the online retreat was useful for planning their program sustainment (mean of 5.75, SD 2.33; range: 0-9), with 9 sites finding it “very useful” (ie, 7 or greater on a 10-point Likert scale). Those with favorable views of the usefulness of the retreat reported that the retreat and other planning activities gave them actionable information to identify and adapt a sustainment strategy. Some sites, however, reported that participation in the retreat and other activities was minimally useful because they already had high confidence in their ability to sustain GRECC Connect program activities. A small number of sites did not rely as heavily on external innovation funding, so they felt the absence of that funding would have minimal impact.

Similarly, sites reported that the sustainment proposal worksheet was useful to plan for their program sustainment (mean of 6.56, SD 2.34; range: 0-10), with 10 sites reporting it was “very useful” (ie, 7 or greater on a 10-point Likert scale). The process of completing the proposal was minimally useful for some sites; however, those sites felt they could already sustain program activities and would not be impacted by a potential shift in funding. Sites that found the proposal activity “very useful” shared that they dedicated time as a team to “sit down” and talk about a sustainment strategy. Although not all sites may have found a concrete solution, staff reported that having time allocated to discussing future sustainment was useful for identifying potential barriers or facilitators to sustaining future program activities. Site-level discussions enabled team members to consider a variety of local options for future sustainment strategies.

Sites were confident that services to rural patients would be sustained using their proposed strategy (mean of 7.38, SD 2.18; range: 3-10), with 12 sites being “highly confident” (ie, 7 or greater on a 10-point Likert scale). One site had low confidence owing to “higher competing demands for resources at their facility.” Another site expressed uncertainty about the proposed shift in funding and was unclear about what the next steps would be, needing more guided support. Many sites that felt “highly confident” shared that they were already well integrated into another hospital service line that could support future program services.

## Discussion

### Principal Findings

Our 3-phase participatory approach—in and of itself a sustainment strategy, and a process for identifying strategies that will sustain the program across 19 sites—serves as an example of how to support maintenance of a large-scale health care program that exists within diverse settings. Given the lack of examples of how to sustain such programs, this is an important contribution to the implementation science literature.

### Strengths of the Approach

In this case example of sustainment support, we found the approach supported broad participation. It intentionally allowed for staff in various roles and professions to engage in the process, learn about the “bigger picture” of the local and national trajectory of the program, share experiences, and voice their ideas and opinions about sustaining GRECC Connect activities. Additionally, this approach enabled sites to distill their thoughts over the course of 3-phases, moving them from a reflection-based response to identifying concrete next steps for program sustainment. Sites had an opportunity to learn from others’ experiences, and a preexisting, strongly supported administrative structure helped facilitate cross-site learning. Many of the activities described above occurred during previously established meeting times, mitigating scheduling barriers and enabling many hub sites and their team members to meaningfully engage with the participatory process.

### Considerations for Future Use of the Approach

Challenges with this approach included balancing direction from the program funder (ie, ORH staff) and leadership (ie, GRECC Connect national director and Core Team members) with the generation of ideas from the group level. While the hub site teams were included in the initial discussions about potential sustainment strategies during monthly all-site meetings before the retreat, the core team leads chose what they felt to be the 3 most promising strategies to discuss in depth at the retreat. This made the retreat discussion more manageable, but may also have unintentionally, prematurely limited the scope of possible sustainment paths discussed and considered by each site. Providing more opportunities for the sites to generate ideas at the local level before hosting the retreat may have mitigated this issue.

Another challenge was that, in trying to adapt to local contexts among the sites, the sustainment worksheet was purposefully not overly specific in the type of responses requested. As the sites interpreted the site sustainment worksheet in their own way, the responses received were wide-ranging in their specificity. This made it difficult to compare across sites and determine next steps for supporting them. More clarity or instructions for how to interpret the worksheets would have been helpful. Additionally, the sustainment planning worksheet was designed to mirror a grant proposal, a format that many clinicians were not familiar with. A more useful format may have been one that encouraged them to develop a business case for their program because the next steps often included convincing leadership of the need to sustain their services.

Finally, throughout the participatory process, the QualEC team offered support to sites at a consistent time and day of the week as an “office hours” format. No sites used that time for support. We found more success inviting participants to ask questions or express concerns during national monthly calls in the months following the online retreat.

### Implications

By describing our approach in detail and highlighting the benefits and challenges, we are responding to a call in implementation science literature [3,9] to provide models for supporting program sustainment. This 3-phase participatory approach is not only an example of dynamic sustainment support but also functions to identify the type of dynamic sustainment support that would be successful in each site’s given context. Wolfenden et al [12] recently highlighted a public health example of dynamic sustainment support called “physically active children in education.” This model of support was used to improve primary schools’ implementation of a mandatory physical activity policy. The pilot study of this approach found that it was effective at increasing teachers’ implementation of the physical activity policy and led to a significant increase in students’ physical activity levels. Like our example, the physically active children in education model was used to sustain a large-scale program that exists in heterogeneous settings and occurred in phases, each successive phase informed by the last, to develop sustainment strategies.

We used our participatory approach during what Nevedal et al [9] consider a “liminal stage” of program sustainment: GRECC Connect is at a crossroads and, given anticipated shifts in funding, can neither be considered sustained nor discontinued. Similar to our findings, Nevedal et al [9] found that among facility representatives, a wide range of perceived optimism about their site’s ability to sustain the evidence-informed practices in question. This underscores the fact that the “liminal stage” of a program’s sustainment is a critical moment in a program’s lifecycle, a moment in which providing additional sustainment support can be particularly beneficial.

Finally, our model makes an important contribution to the literature in that it uses a participatory approach to address the reality that successful sustainment strategies will likely look different for each of the 19 sites where GRECC Connect is implemented. To best support them, we must learn about each site’s local barriers and facilitators. At the same time, a large-scale program has cumulative knowledge and a broad perspective that enhances local staff’s capacity to plan for sustainment. This participatory approach may be of value to other large-scale health care programs with established administrative and organizational structures to support cross-site information exchange and significant site-level participation.

### Limitations

This description of our 3-phase participatory approach to program sustainment has limitations. First, we have not had the opportunity to assess the impact of this approach and therefore cannot yet speak to its efficacy in sustaining clinical services. As the dynamic sustainment support continues to be adapted to support GRECC Connect programming, future research will reveal if sites were able to maintain access to geriatric services for older patients in rural areas over the long term. Additionally, the generalizability of our approach may be limited insofar as other clinical programs may not have the robust administrative structure needed to help guide this 3-phase participatory approach.

### Conclusions

The sustainment of EBPs, such as GRECC Connect, is an important goal to help avoid the costs and burdens of implementing new, similar programs. Without established methods to support the sustainment of these programs, implementation science continues to lack concrete models of how to place frameworks into practice. In this participatory case example, we identify processes and tools to identify appropriate sustainment strategies for a large-scale, national health care program that exists in heterogeneous settings. While more work is needed to understand its efficacy, our approach serves as an innovative example of a dynamic sustainment support strategy conducted in the liminal stage of program sustainment.

## Appendix

Multimedia Appendix 1 GRECC Connect retreat breakout room discussion questions.

Multimedia Appendix 2 GRECC Connect site sustainment planning worksheet.

## Abbreviations

CRH: Clinical Resource Hub
EBP: evidence-based program
GEC: Geriatrics and Extended Care
GeriPACT: Geriatric Patient Aligned Care Team
GRECC: Geriatric Research, Education, and Clinical Centers
ORH: Office of Rural Health
PSAT: Program Sustainability Assessment Tool
QualEC: GRECC Connect Qualitative Evaluation Core team
RE-AIM: Reach, Effectiveness, Adoption, Implementation, and Maintenance
VA: Department of Veterans Affairs

## Data Availability

The datasets generated or analyzed during this study are available from the corresponding author on reasonable request.
