# Supplementary material for: Participatory Approach to Program Sustainment: Example From a Multisite National Geriatrics Telemedicine Program
Source: JMIR Form Res. 2026 Mar 27;10:e82409. doi: 10.2196/82409 (PMC13069370; doi:10.2196/82409)
Supplement: Multimedia Appendix 1 [file formative_v10i1e82409_app1.pdf]

GRECC Connect Post ORH Site Visit Retreat
Small Group Discussion Guide

GUIDELINES FOR Small Group Discussion Format: TOTAL time 20 minutes

- **Identify Reporter** (Person will summarize discussion when small groups reconvene)
- **Identify Notetaker** (Person will write notes)

- **Discussion facilitators** (Person(s) guiding discussion)
  - Ensure EVERYONE in group has chance to speak
    - Consider limiting time (<1 min per person)
    - Consider only allowing person to speak again after everyone else has had a chance to speak
    - No comment or question is a bad one
  - Keep discussion focused on priority question(s) tasked

- We will give ½ way 20-, 10-, & 5- minute warnings for small group time to wrap-up

GRECC Connect Post ORH Site Visit Retreat
Small Group Discussion Guide

	**How important is this strategy? Why or why not?**
	**What could working with GeriPACT/GEC look like? (list scenarios)**
	**What are barriers to working with GeriPACT/ GEC?**<br>**What are factors that may facilitate working with GeriPACT/GEC?**

Other comments/ notes	
