# Supplementary material for: Participatory Approach to Program Sustainment: Example From a Multisite National Geriatrics Telemedicine Program
Source: JMIR Form Res. 2026 Mar 27;10:e82409. doi: 10.2196/82409 (PMC13069370; doi:10.2196/82409)
Supplement: Multimedia Appendix 2 [file formative_v10i1e82409_app2.pdf]

GRECC Connect
Site-Specific Sustainment Strategies to Serve Rural, Older Veterans
Planning Worksheet for FY24-25 Transition

Site:

Project Proposal *(1.5-page limit. Can be bullet format)*

I. **Background and rural significance** *(What is the need for this service/program at your site, as it relates to rural health? [e.g., rural Veteran population, existing relationships with rural CBOCs])*

II. **Purpose and objectives** *(How does your program fulfill the need you identified above?)*

III. **Design and planned activities** *(from FY24 prep activities through FY25 and beyond main activities, including staffing, timeline, etc.)*

IV. **Key partners** *(e.g., local or VISN-level service line managers, leadership, CRH director)*

V. **Measurement of key rural Veteran outcomes**

VI. **Products and deliverables needed to make your case for sustainment to your Facility/VISN Audience** *(e.g., what data do you need to show your facility or VISN leadership to ensure ongoing support for your program in FY25 and beyond?)*

VII. **Long-term plan and potential for growth and/or sustainment**

VIII. **Potential barriers and how you might address them**

<u>Reflections for GRECC Connect Qualitative Core Team:</u> *(Please consider these questions at your site level, as appropriate)*

I. **A. To what extent was your <u>participation</u> in the March 2023 Virtual GRECC Connect Retreat, as well as other site planning activities (discussions with other sites regarding sustainment strategies, self-assessment tools [PSAT]), helpful for your sites' planning?** (Rate: 0 [not at all helpful] - –0 [extremely helpful])

Not helpful at all										Extremely Helpful
⓪	①	②	③	④	⑤	⑥	⑦	⑧	⑨	⑩
☐	☐	☐	☐	☐	☐	☐	☐	☐	☐	☐

B. Please explain.

II. **A. To what extent was the process of completing <u>this proposal</u> helpful for your sites' planning?** (Rate: 0 [not at all helpful] - 10 [extremely helpful])

Not helpful at all										Extremely Helpful
⓪	①	②	③	④	⑤	⑥	⑦	⑧	⑨	⑩
☐	☐	☐	☐	☐	☐	☐	☐	☐	☐	☐

B. Please explain.

III. **A. How confident are you that your service to rural Veterans can be sustained using your proposed site sustainment strategy?** (Rate: 0 [not confident at all] – 10 [totally confident])

Not confident at all										Totally Confident
⓪	①	②	③	④	⑤	⑥	⑦	⑧	⑨	⑩
☐	☐	☐	☐	☐	☐	☐	☐	☐	☐	☐

B. Please explain.

IV. **Are there additional things that GRECC Connect leadership could do to support your sites' sustainment planning?**
